# Population-scale cross-disorder atlas of the human prefrontal cortex at single-cell resolution

**DOI:** 10.1038/s41597-025-04687-5

**Published:** 2025-06-06

**Authors:** John F. Fullard, Prashant NM, Donghoon Lee, Deepika Mathur, Karen Therrien, Aram Hong, Clara Casey, Zhiping Shao, Marcela Alvia, Stathis Argyriou, Tereza Clarence, David Burstein, Sanan Venkatesh, Pavan K. Auluck, Lisa L. Barnes, David A. Bennett, Stefano Marenco, PsychAD Consortium, John F. Fullard, Prashant NM, Donghoon Lee, Aram Hong, Clara Casey, Zhiping Shao, Marcela Alvia, Stathis Argyriou, Tereza Clarence, David Burstein, Pavan K. Auluck, Lisa L. Barnes, David A. Bennett, Stefano Marenco, Monika Ahirwar, Sayali A. Alatkar, Marios Anyfantakis, Rachel Bercovitch, Pramod B. Chandrashekar, Jerome Choi, Noah Cohen Kalafut, Pengfei Dong, Logan C. Dumitrescu, Steven Finkbeiner, Chirag Gupta, Kalpana H. Arachchilage, Chenfeng He, Timothy J. Hohman, Xiang Huang, Lars J. Jensen, Ting Jin, Pavel Katsel, Saniya Khullar, Seon Kinrot, Steven P. Kleopoulos, Roman Kosoy, Mikaela Koutrouli, Athan Z. Li, Nicolas Y. Masse, Deepika Mathur, Colleen A. McClung, Jennifer Monteiro Fortes, Milos Pjanic, Christian Porras, Vivek G. Ramaswamy, Genadi Ryan, Madeline R. Scott, Lyra Sheu, Maxim Signaevsky, Collin Spencer, Karen Therrien, Fotios Tsetsos, Sanan Venkatesh, Daifeng Wang, Xinyi Wang, Zhenyi Wu, Hui Yang, Biao Zeng, Kiran Girdhar, Vahram Haroutunian, Gabriel E. Hoffman, Georgios Voloudakis, Jaroslav Bendl, Panos Roussos, Kiran Girdhar, Vahram Haroutunian, Gabriel E. Hoffman, Georgios Voloudakis, Jaroslav Bendl, Panos Roussos

**Affiliations:** 1 Center for Disease Neurogenomics, Icahn School of Medicine at Mount Sinai, New York, NY USA; 2 Friedman Brain Institute, Icahn School of Medicine at Mount Sinai, New York, NY USA; 3 Department of Psychiatry, Icahn School of Medicine at Mount Sinai, New York, NY USA; 4 Department of Genetics and Genomic Sciences, Icahn School of Medicine at Mount Sinai, New York, NY USA; 5 Mental Illness Research Education and Clinical Center (VISN 2 South), James J. Peters VA Medical Center, Bronx, NY USA; 6 Center for Precision Medicine and Translational Therapeutics, James J. Peters VA Medical Center, Bronx, NY USA; 7 Graduate School of Biomedical Science, Icahn School of Medicine at Mount Sinai, New York, NY 10029, USA, New York, NY 10029 USA; 8 Human Brain Collection Core, National Institute of Mental Health-Intramural Research Program, Bethesda, MD USA; 9 Rush Alzheimer’s Disease Center, Rush University Medical Center, Chicago, Illinois USA; 10 Department of Neurological Sciences, Rush University Medical Center, Chicago, Illinois USA; 11 Department of Neuroscience, Icahn School of Medicine at Mount Sinai, New York, NY USA; 12 Center for Systems and Therapeutics, Gladstone Institutes, San Francisco, CA USA; 13 Taube/Koret Center for Neurodegenerative Disease Research, Gladstone Institute, San Francisco, CA USA; 14 Waisman Center, University of Wisconsin-Madison, Madison, WI USA; 15 Department of Computer Sciences, University of Wisconsin-Madison, Madison, WI USA; 16 Department of Biostatistics and Medical Informatics, University of Wisconsin-Madison, Madison, WI USA; 17 Department of Population Health Sciences, University of Wisconsin-Madison, Madison, WI USA; 18 Vanderbilt Memory & Alzheimer’s Center, Vanderbilt University Medical Center, Nashville, TN USA; 19 Vanderbilt Genetics Institute, Vanderbilt University Medical Center, Nashville, TN USA; 20 Department of Neurology, University of California San Francisco, San Francisco, CA USA; 21 Department of Physiology, University of California San Francisco, San Francisco, CA USA; 22 Neuroscience and Biomedical Sciences Graduate Programs, University of California San Francisco, San Francisco, CA USA; 23 Novo Nordisk Foundation Center for Protein Research, Faculty of Health and Medical Sciences, University of Copenhagen, Copenhagen, Denmark; 24 Department of Psychiatry, University of Pittsburgh School of Medicine, Pittsburgh, PA USA

**Keywords:** Gene expression, Neurodevelopmental disorders, Neurological disorders

## Abstract

Neurodegenerative diseases and serious mental illnesses often exhibit overlapping characteristics, highlighting the potential for shared underlying mechanisms. To facilitate a deeper understanding of these diseases and pave the way for more effective treatments, we have generated a population-scale multi-omics dataset consisting of genotype and single-nucleus transcriptome data from the prefrontal cortex of frozen human brain specimens. Encompassing over 6.3 million nuclei from 1,494 donors, our dataset represents a diverse range of neurodegenerative and serious mental illnesses, including Alzheimer’s and Parkinson’s diseases, schizophrenia, bipolar disorder and diffuse Lewy body dementia, as well as neurotypical controls. Our dataset offers a unique opportunity to study disease interactions, as 21% of donors had comorbid diagnoses of two or more major brain disorders. Additionally, it includes detailed phenotypic information on neuropsychiatric symptoms, such as apathy and weight loss, which commonly accompany Alzheimer’s disease and related dementias. We have performed stringent preprocessing and quality controls, ensuring the reliability and usability of the data. As a commitment to fostering collaborative research, we provide this valuable resource as an online repository, enabling widespread analyses across the scientific community.

## Background & Summary

Alterations in gene expression and changes in cell type abundances are commonly observed in various brain-related disorders, ranging from mental illnesses like schizophrenia (SCZ)^1,2^ to neurodegenerative diseases such as Alzheimer’s (AD)^3–5^ and Parkinson’s disease (PD)^6,7^. Traditional methods, using bulk tissue or broad populations of sorted cells, fail to fully capture the intricate, often highly cell type specific, molecular changes associated with these diseases. Recent advances in single-cell expression profiling address these limitations and have facilitated the generation of larger datasets, most notably for AD where the combined data now nears 1,000 cases^3,8–11^. However, single cell resolution datasets for other diseases are considerably smaller. For instance, the latest release from PsychENCODE, which consolidates all major sources of single-cell data on serious mental illnesses, reported only 77 cases of SCZ, 52 cases of autism, 34 cases of bipolar disorder (BD), and 10 cases of post-traumatic stress disorder^2,12^. Although the existing datasets offer valuable insights into each disorder separately, the potential for conducting complex analyses across different disorders to identify shared or distinct molecular pathways is still limited. This is mainly due to the small sample sizes and challenges caused by merging studies generated by different research groups, introducing an additional layer of systematic bias.

**Fig. 1:**
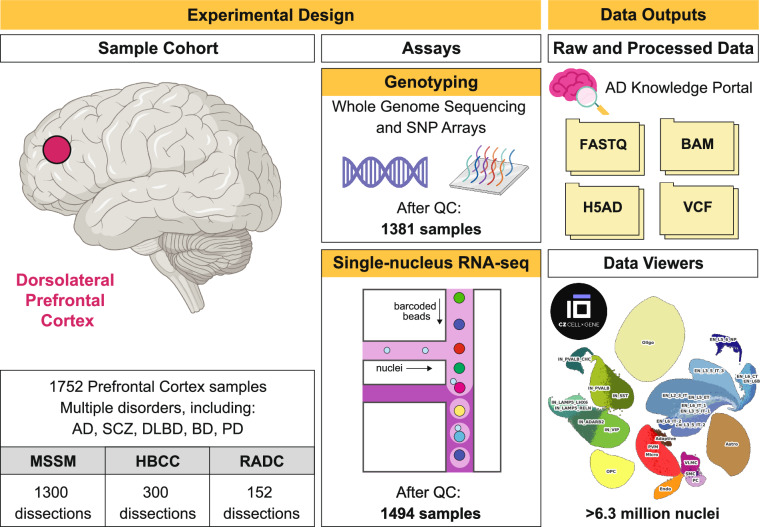
Overview of the dataset collection process and key outputs of the study.

To enhance our ability to identify shared and distinct molecular pathways, causal variants, and genes involved in various brain-related disorders, we generated the largest collection, to date, of single-nucleus gene expression data in the human brain (Fig. 1). This collection comprises over 6.3 million individual nuclei, isolated from 1,494 frozen postmortem samples of the dorsolateral prefrontal cortex (DLPFC). We selected the DLPFC for our study due to its critical role in higher-level brain processes, including cognitive function, decision making, and emotional regulation, all of which are often impaired in the diseases under investigation^13,14^. Importantly, the DLPFC has been implicated in numerous neuroimaging and neuropathological studies as a region that undergoes significant pathological changes in both neurodegenerative diseases and serious mental illnesses^15–17^.

The sample cohort consists of neurotypical controls, as well as donors affected by more than 30 different disorders, including three represented by more than 100 cases (AD (n = 519), SCZ (n = 177), and diffuse Lewy Body disease (DLBD; n = 112)) and three by more than 40 (vascular dementia (n = 85), BD (n = 72) and PD (n = 48)). In addition to providing a more detailed characterization of these somewhat well-studied diseases, our dataset also includes a subset of cases with relatively understudied conditions, such as obsessive-compulsive disorder (n = 6), amyotrophic lateral sclerosis (n = 5), progressive supranuclear palsy (n = 5), argyrophilic grain disease (n = 3) or normal pressure hydrocephalus (n = 3). To our knowledge, many of these disorders have not yet been analyzed at the single-cell level. Despite the small number of cases, analyzing them alongside well-matched controls for sex, age, and race could offer valuable preliminary insights into these conditions. Lastly, we want to highlight the availability in our cohort of phenotypic information on neuropsychiatric symptoms (NPS), which frequently accompany AD and related dementias^18^. Throughout the course of the disease, more than 80% of individuals with AD are estimated to exhibit at least one NPS that significantly impacts their clinical outcomes^19^. So far, various studies have examined population data to characterize NPS along the AD continuum^20–22^. For example, depression and apathy are often the most observed symptoms in the early stages of AD, with delusions, hallucinations, and aggression becoming more prevalent as the disease advances^20^. Yet, beyond broad population-level observations, research into the mechanistic basis of these NPS remains scarce. We believe that our dataset provides a unique opportunity to explain NPS in AD at a more granular level, potentially leading to a better understanding of the disease and the identification of novel therapeutic targets.

The release of this dataset by the PsychAD consortium is accompanied by a series of manuscripts describing the cross-disorder analysis of transcriptomic vulnerability^23^, genetic regulation of gene expression^24^ and transcriptome-wide association studies^25^. The consortium has also leveraged neurotypical controls to assemble a map of transcriptomic changes across the lifespan^26^. Lastly, the computational scale and diversity of the generated data led to the development of analytical tools and databases, including *dreamlet* for differential gene expression^27^, *PASSCODE* for detecting phenotype-associated cells^28^ and *iBrainMap* for personalized functional genomics analysis, enabling the identification of cell-type-specific regulatory networks and phenotypic prioritization^29^.

## Methods

### Cohort data collection

**Fig. 2:**
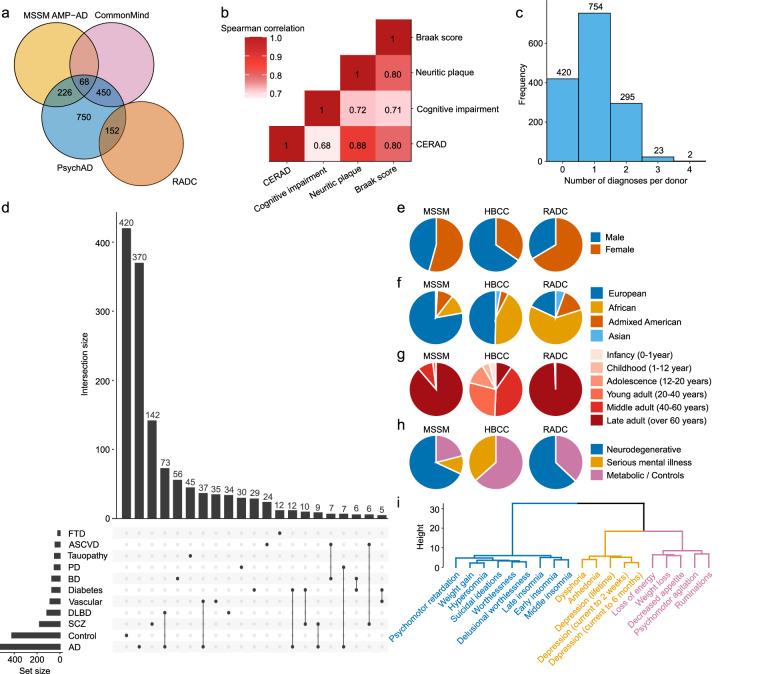
Summary of demographics and clinical data of the PsychAD cohort. (**a**) Overlap of the PsychAD cohort with MSSM AMP-AD, CommonMind and RADC cohorts. (**b**) Correlations among AD-related phenotypes. This analysis includes donors with either sole AD diagnosis (without comorbidities) or control samples (free of any diagnosis). For the “cognitive impairment” phenotype, untransformed CDR values are used for MSSM donors. RADC donors are numerically scaled as follows: Mild Cognitive Impairment (MCI) = 0.75, clinical dementia = 3. (**c**) Distribution of the number of diagnoses per donor. Note that “Dementia” and “MCI” are not counted as separate diagnoses if the donor already has a neurodegenerative or neurological disease. Also, NPS are excluded from this comparison. (**d**) Analysis of the counts and intersections among the top 10 most frequently represented diagnoses plus controls, with a minimum intersection size for plotting set to 10. FTD: Frontotemporal dementia; ASCVD: Atherosclerotic cardiovascular disease; PD: Parkinson’s disease; BD: Bipolar disorder; Diabetes: Diabetes mellitus Type 1/2/unspecified; Vascular: Vascular dementia; DLBD: Diffuse Lewy body disease; SCZ: Schizophrenia. (**e**–**h**) Exploration of demographic and clinical variables within subcohorts of samples from the three brain tissue sources, encompassing sex (**e**), genetically inferred ancestry (**f**), age (**g**), and disease status (**h**). NPS are not included in the disease count in (**h**). (**i**) Dendrogram of NPS based on their co-occurrence with three highlighted clusters.

The “PsychAD cohort” comprises 1,494 donors, all of whom have undergone single nucleus RNA-seq (snRNA-seq) analysis. Among these donors, genotype data is available for 1,381 (92%) of them. Specimens came from multiple sources, the Mount Sinai NIH Brain Bank and Tissue Repository (MSSM; 1,042 samples), the NIMH-IRP Human Brain Collection Core (HBCC; 300 samples), and five prospective cohort studies at the Rush Alzheimer’s Disease Center (RADC; 152 samples)^30,31^ (Fig. 2a). All five RADC cohorts were approved by an Institutional Review Board of Rush University Medical Center and participants signed informed and repository consents and an Anatomic Gift Act for organ donation. Importantly, 60% of the PsychAD cohort, totaling 896 donors, had previously been included in MSSM AMP-AD^32^, CommonMind^33^ and/or RADC studies^34^, which had already generated a wealth of omics data for these individuals, including SNP-array^33^, whole-genome sequencing (WGS)^32^, RNA sequencing (RNA-seq)^35–37^, assay for transposase-accessible chromatin (ATAC-seq)^4,7,35,38^, DNA methylation^37^, proteomics^35^, chromatin immunoprecipitation sequencing for histone 3 lysine 27 acetylation (ChIP-seq H3K27ac)^4,39^ and for histone 3 lysine 4 trimethylation (ChIP-seq H3K4me3). However, it is important to emphasize that, prior to this study, no snRNA-seq data was available for the PsychAD cohort, with the exception of 53 donors from MSSM^2^ and 7 from RADC^3,11^.

Because the institutions provided the donor’s clinical records in different formats, our first imperative was to harmonize the diagnosis status prior to downstream analyses. The full sample cohort captures 20 neurodegenerative/neurologic diseases (e.g. AD, PD, DLBD), 13 serious mental illnesses (e.g. SCZ, BD), 19 NPS (e.g. insomnia, weight loss) and 4 metabolic diseases (e.g. Type 1/2 diabetes) (Table 1). The presence of these diseases is typically encoded in a binary format, except for AD, for which we have: (1) case-control status defined using the Consortium to Establish a Registry for Alzheimer’s Disease (CERAD) criteria^40^; (2) Braak AD-staging score for progression of neurofibrillary neuropathology^15,41^; (3) mean density of neuritic plaques (plaque mean); and (4) assessment of dementia and cognitive status based on clinical dementia rating scale (CDR) for MSSM samples^42^, or final summary clinical diagnosis (cogdx) for RADC^43^. For binary diagnosis classification within the PsychAD dataset, we define the AD category as follows: CERAD ≥ 2, Braak ≥ 3 and CDR ≥ 1/cogdx ≥ 4 (MSSM/RADC). Donors with a neuropathological burden but no cognitive loss are categorized as “Tauopathy” (CERAD = 1, Braak ≥ 3 for both MSSM and RADC and, additionally, CDR = 0 for MSSM/no cognitive impairment for RADC) (Tables 1, 2). Depending on the severity of cognitive loss, the donors are classified as Mild Cognitive Impairment (MCI; CDR = 0.5 for MSSM; MCI for RADC) or Dementia (CDR ≥ 1 for MSSM; clinical dementia for RADC) (Tables 1, 2). The AD-related neuropathological and clinical phenotypes are moderately correlated (Fig. 2b), indicating shared and distinct disease processes^4^. While the PsychAD cohort contains 420 donors with no diagnosis (28% of the dataset, referred to as “control” samples) and 754 donors with exactly 1 diagnosis (51% of the dataset), the remaining 320 donors (21%) are associated with 2 or more diagnoses (Fig. 2c,d). The demographics and clinical characteristics of donors varied significantly among the sources (Fig. 2e–h), with donors of European ancestry constituting over 79% of the MSSM subcohort, compared to 50% in HBCC, and 18% in RADC (Fig. 2f). This variation enables the exploration of ancestry-specific disease signatures. Regarding age distribution, 89% and 99% of individuals in the MSSM and RADC subcohorts are over 60 years old, respectively, while only 10% of HBCC are among this older age category, with 49% of donors under the age of 40 (Fig. 2g). These age distribution patterns partly align with disease distribution, as MSSM and RADC primarily consist of donors with or at risk for neurodegenerative/neurological diseases, for which age is a major risk factor (Fig. 2h). In contrast, in addition to controls, HBCC exclusively includes serious mental illnesses, which typically manifest during childhood or adolescence^44–47^. Due to HBCC’s different disease focus, we lack certain AD-related metrics (CERAD, BRAAK, CDR, Plaque Mean) that are available for MSSM and RADC. However, HBCC’s sample selection process involved reviewing neuropathology reports to ensure the absence of significant plaque and/or tangle pathology. As a result, HBCC donors without brain-related diagnoses can reliably be used as controls for comparison with neurodegenerative diseases, even in the absence of additional neuropathological data.

**Table 1:** Summary of all diagnoses recognized within the PsychAD cohort.

Disease	Category^a^	Sample size				Age of death (mean)		
		Total	MSSM	HBCC	RADC	MSSM	HBCC	RADC
Dementia^b^	NDD	752	666	0	86	82.7		85.1
Alzheimer’s disease^c^	NDD	519	447	0	72	84.3		85.8
Schizophrenia	SMI	177	127	50	0	72.1	52.1	
Mild cognitive impairement^d^	NDD	125	103	0	22	77.4		83.4
Diffuse Lewy body disease	NDD	112	107	0	5	82.1		80.7
Vascular dementia	NDD	85	65	0	20	82.7		83.9
Bipolar disorder type I	SMI	55	0	55	0		43.7	
Tauopathy^e^	NDD	45	33	0	12	87.6		84.1
Diabetes mellitus unspecified	MD	42	0	1	41		75.0	87.9
Atherosclerotic cardiovascular disease	MD	40	0	40	0		48.1	
Parkinson’s disease	NDD	40	25	0	15	81.8		80.3
Type 1 diabetes	MD	16	0	16	0		55.6	
Frontotemporal dementia	NDD	16	15	0	1	78.1		67.4
Neuroleptic-induced tardive dyskinesia	SMI	14	0	14	0		53.0	
Head injury	NDD	12	6	0	6	68.3		85.2
Seizures	NDD	12	0	12	0		48.2	
Type 2 diabetes	MD	11	0	11	0		53.7	
Encephalitis, uncertain Parkinson’s disase	NDD	8	8	0	0	79.1		
Brain tumor	NDD	8	7	1	0	75.3	45.0	
Cerebral atrophy	NDD	7	7	0	0	71.0		
Bipolar disorder type II	SMI	7	0	7	0		43.7	
Amyotrophic lateral sclerosis	NDD	6	5	0	1	79.0		53.8
BD NOS (not otherwise specified)	SMI	6	0	6	0		35.7	
Obsessive-compulsive disorder	SMI	6	0	6	0		47.5	
Progressive supranuclear palsy	NDD	5	5	0	0	80.6		
Leucotomy/Lobotomy	NDD	4	4	0	0	79.8		
Anorexia nervosa	SMI	4	0	4	0		42.8	
Schizoaffective bipolar disorder	SMI	4	0	4	0		45.8	
Argyrophilic grain disease	NDD	3	3	0	0	83.0		
Multiple sclerosis	NDD	3	3	0	0	41.7		
Normal pressure hydrocephalus	NDD	3	3	0	0	83.0		
Bulimia nervosa	SMI	3	0	3	0		47.0	
Major depressive disorder	SMI	3	2	1	0	62.5	55.0	
Epilepsy	NDD	2	0	2	0		64.0	
Attention deficit hyperactivity disorder	SMI	2	0	2	0		32.5	
Post-traumatic stress disorder	SMI	2	1	1	0	60.0	44.0	
Schizoaffective depressive disorder	SMI	2	0	2	0		42.5	

^a^NDD: Neurodegenerative/neurological disease; SMI: serious mental illness; MD: metabolic disease.

^b^MSSM: derived from Clinical Dementia Score (CDR ≥ 1); RADC: derived from Consensus Cognitive Status (cogdx ≥ 4).

^c^MSSM: derived from (CERAD ≥ 2 and Braak ≥ 3 and CDR ≥ 1); RADC: derived from (CERAD ≥ 2 and Braak ≥ 3 and cogdx ≥ 4).

^d^MSSM: derived from Clinical Dementia Score (CDR == 0.5); RADC: derived from Consensus Cognitive Status(cogdx ∈ (2,3)).

^e^MSSM: defined as (CERAD == 1 and Braak ≥ 3 and CDR == 0); or determined by brain bank; RADC: defined as (CERAD == 1 and Braak ≥ 3 and cogdx == 1).

**Table 2:** Classification of donors by various clinical and neuropathological measurements related to AD diagnosis.

AD phenotype		Sample size			Age of death (mean)	
Name	Value	Total	MSSM	RADC	MSSM	RADC
CERAD^a^	1	341	308	33	70.6	81.6
CERAD^a^	2	113	103	10	84.6	81.7
CERAD^a^	3	229	189	40	88.2	87.3
CERAD^a^	4	394	325	69	81.4	84.5
BRAAK^b^	0	163	156	7	59.7	70.2
BRAAK^b^	1	107	102	5	75.8	75.8
BRAAK^b^	2	161	149	12	81.2	76.7
BRAAK^b^	3	161	135	26	84.5	85.0
BRAAK^b^	4	122	75	47	86.9	88.0
BRAAK^b^	5	134	92	42	87.1	87.9
BRAAK^b^	6	328	315	13	82.6	77.5
Plaque mean^c^	0	384	384	0	70.2	
Plaque mean^c^	0.01–4.89	158	158	0	86.0	
Plaque mean^c^	4.90–8.72	158	158	0	85.3	
Plaque mean^c^	8.73–14.03	158	158	0	85.9	
Plaque mean^c^	>14.04	157	157	0	79.7	
MSSM: CDR^d^	0	221	221	0	68.0	
MSSM: CDR^d^	0.5	103	103	0	77.4	
MSSM: CDR^d^	1	83	83	0	80.7	
MSSM: CDR^d^	2	103	103	0	82.6	
MSSM: CDR^d^	3	249	249	0	83.1	
MSSM: CDR^d^	4	111	111	0	84.4	
MSSM: CDR^d^	5	120	120	0	81.6	
RADC: cogdx^e^	1	38	0	38		83.1
RADC: cogdx^e^	2	22	0	22		83.4
RADC: cogdx^e^	4	68	0	68		86.0
RADC: cogdx^e^	5	14	0	14		82.7
RADC: cogdx^e^	6	4	0	4		77.1

^a^CERAD (Consortium to Establish a Registry for Alzheimer’s Disease): Qualitative variable from neuropathological scoring where 1 = normal, 2 = possible AD, 3 = probable AD, 4 = definite AD.

^b^Braak: Braak neurofibrillary tangle score from the regional patterns of the density of neurofibrillary tangles across the brain.

^c^Plaque mean: The average density of neuritic plaque across five brain regions, i.e., middle frontal gyrus, orbital frontal cortex, superior temporal gyrus, inferior parietal lobule and occipital cortex. Plaque categories are defined by quartile values calculated on distribution of non-zero plaque values.

^d^MSSM CDR: Clinical dementia rating available only for MSSM donors where 0 = no dementia, 0.5 = questionable dementia (very mild), 1 = mild dementia, 2 = moderate dementia, 3 = severe dementia, 4 = profound dementia, 5 = terminal dementia.

^e^RADC: cogdx: Final consensus cognitive diagnosis available only for RADC donors where 1 = no cognitive impairment, 2 = MCI but no other CI (cognitive impairment), 3 = MCI and another cause of CI, 4 = AD dementia but no other CI, 5 = AD dementia and another cause of CI, 6 = Other dementia.

In addition to offering disease-related phenotypes, we included a set of 19 NPS, each of which affected between 23 and 438 individuals from the 1,042 MSSM donors. These symptoms constitute commonly associated features of AD and related dementias, and are linked to significant adverse effects on daily function and quality of life^48^. Utilizing hierarchical clustering analysis, we observed that these NPS tend to group into three distinct clusters, broadly aligning with established associations (Fig. 2h). Therefore, as an alternative to analyzing the 19 individual classes, we also offer a categorization of donors into the three aggregated NPS classes (Table 3).

**Table 3:** Summary of the donor counts with defined neuropsychiatric symptoms.

Neuropsychiatric symptom	Type	NPS-present	NPS-absent
Group 1: Early insomnia	specific	93	676
Group 1: Middle insomnia	specific	73	694
Group 1: Late insomnia	specific	91	682
Group 1: Hypersomnia	specific	23	766
Group 1: Weight gain	specific	41	761
Group 1: Suicidal ideations	specific	91	717
Group 1: Delusional worthlessness	specific	53	746
Group 1: Worthlessness	specific	67	730
Group 1: Psychomotor retardation	specific	88	723
Group 1: Sleep/WeightGain/Guilt/Suicide	aggregated	316	519
Group 2: Weight loss	specific	401	401
Group 2: Decreased appetite	specific	415	386
Group 2: Psychomotor agitation	specific	438	390
Group 2: Loss of energy	specific	438	379
Group 2: Ruminations	specific	391	428
Group 2: WeightLoss/PMA	aggregated	678	160
Group 3: Dysphoria	specific	410	408
Group 3: Anhedonia	specific	357	454
Group 3: Depression: current to 2 weeks	specific	254	558
Group 3: Depression: current to 6 months	specific	268	541
Group 3: Depression: lifetime	specific	251	528
Group 3: Depression/Mood	aggregated	448	386

For “aggregated” types of symptoms, a donor is classified as “NPS-present” if they exhibit at least one of the “specific” symptoms. Conversely, a donor is classified as “NPS-absent” if at least one “specific” symptom is marked “false” and no symptoms are marked “true.”

### snRNA-seq data generation and analysis

#### Nuclei isolation and snRNA-seq library preparation

All buffers were supplemented with RNAse inhibitors (Takara). 6 samples were processed in parallel. 25 mg of frozen postmortem human brain tissue from each specimen was homogenized in cold lysis buffer (0.32 M Sucrose, 5 mM CaCl_2_, 3 mM Magnesium acetate, 0.1 mM, EDTA, 10 mM Tris-HCl, pH8, 1 mM DTT, 0.1% Triton X-100) and filtered through a 40 µm cell strainer. The flow-through was underlaid with sucrose solution (1.8 M Sucrose, 3 mM Magnesium acetate, 1 mM DTT, 10 mM Tris-HCl, pH8) and centrifuged at 107,000 xg for 1 hour at 4 °C. Pellets were resuspended in PBS and quantified (Countess II, Life Technologies). 2 M nuclei from each sample were then pelleted at 500 xg for 10 minutes at 4 °C and re-suspended in 100 µl staining buffer (2% BSA, 0.02% Tween-20, 10 mM Tris, 146 mM NaCl, 1 mM CaCl_2_ and 21 mM MgCl). Each sample was incubated with 1 µg of a distinct TotalSeq-A nuclear hashing antibody (Biolegend) for 30 min at 4 °C. Prior to fluorescence activated nuclei sorting (FANS), volumes were brought up to 250 µl with staining buffer and 7-AAD (Invitrogen) added to facilitate the detection of nuclei. 7-AAD positive nuclei were sorted into tubes pre-coated with 5% BSA using a FACSAria flow cytometer (BD Biosciences).

Following FANS, nuclei were washed in staining buffer before being re-suspended in 22 µl PBS and quantified. Nuclei concentrations were normalized and equal amounts from each sample were pooled together. 2 aliquots of 60,000 pooled nuclei (i.e. 10,000 per sample) were processed in parallel using 3′ v3.1 reagents (10x Genomics). At the cDNA amplification step (step 2.2), reactions were supplemented with a hash-tag oligo (HTO) cDNA “additive” primer (GTGACTGGAGTTCAGACGTGTGCTCTTCCGAT*C*T; *Phosphorothioate bond). Following cDNA amplification, supernatants from the 0.6x SPRI selection step were retained for HTO library generation. Otherwise, cDNA libraries were prepared according to the manufacturer’s instructions (10x Genomics). HTO libraries were prepared as described previously^49^

#### Computational processing

Sequencing reads from all pools of multiplexed samples were aligned to the hg38 reference genome using STARsolo^50,51^. To assign the nuclei from each pool to their respective donors, we applied a genotype-based demultiplexing approach followed by a genotype concordance check. First, cellSNP^52^ was used to pile up the alleles from polymorphic sites overlapping snRNA-seq reads within expressed genes (for inclusion, a gene needed to be expressed by at least 10 cells). Polymorphic sites had to show a minimum minor allele frequency of 0.1 and a minimum aggregated UMI count of 20. Then, vireo^53^ utilized those pile-ups to split cells into clusters corresponding to six distinct donors per pool. The assignment of the identity of each cluster of cells to a particular donor was derived from genotype concordance analysis that compared the clusters of cells against reference genotyping data using QTLtools-mbv^54^. This analysis could be accurately performed only for cells exceeding baseline quality control (QC) metrics, i.e. minimum number of expressed genes (n ≥ 1,000) and maximum fraction of mitochondrial reads (less than 5%). Cells that didn’t meet these criteria were excluded. While the majority of pools contained the cells from the expected sets of donors, we leveraged the genotype concordance results to detect and correct occasional sample swaps and mislabeling.

After genome alignment and demultiplexing, the downstream processing was performed using Pegasus and scanpy^55^. We applied rigorous three-step QC to remove ambient RNA and retain viable cells for downstream analysis. First, we implemented a more stringent QC for individual cells, in addition to the initial QC carried out during the demultiplexing stage. Cells falling outside the defined ranges for UMI counts (1,500 ≤ n_UMIs ≤ 110,000), gene counts (1,100 ≤ n_genes ≤ 12,500), and mitochondrial content (less than 5%) were removed. We also checked for possible contamination from ambient RNA using CellBender^56^. Further filtering was carried out by removing doublets using the Scrublet method^57^. Second, the QC was applied at the feature level. We removed features (genes) that were not robustly expressed by at least 0.05% of the nuclei. Lastly, the QC was applied at the donor level and, because they could introduce noise in downstream analysis, those with less than 50 nuclei were removed. Then, to correct for unwanted (non-biological) variance, such as dissection biases arising from differing tissue source protocols across source brain banks, we employed Canonical Correlation Analysis using the Harmony method^58^. Highly variable features were selected from mean and variance trends, and we used the k-nearest-neighbor (kNN) graph calculated on the basis of harmony-corrected PCA embedding space to cluster cells in the same cell type using Leiden^59^ clustering algorithms. We used UMAP (Uniform Manifold Approximation and Projection)^60^ for the visualization of the resulting clusters.

#### Defining cellular taxonomy using iterative clustering

Cellular taxonomy was defined using a divide-and-conquer strategy. From the full dataset containing over 6 million nuclei, 8 major cell classes were defined as described above. After subsetting the data by each class, we re-calculated highly variable genes (HVGs) among cells in the same class. This allowed us to re-focus on feature space that is more relevant for the same class of cells. We then calculated kNN graph on the basis of the harmony-corrected PCA of the selected HVGs. Leiden-clustering was used to annotate subclass-level annotation. We iterated to the second level of taxonomy which returned us with 67 subtypes of human brain cell types.

### Genotyping

#### Overall strategy

The majority of donors from the HBCC (98%) and RADC (87%) had previously undergone genotyping^32,33,61^ and we opted to utilize this existing data. For the MSSM donors, however, the coverage from previous genotyping efforts was notably lower, with only 57% (598 donors) having been genotyped. Therefore, we conducted genotyping for all MSSM donors for whom we had a sufficient amount of material, as detailed in the “Library preparation for MSSM donors” and “Computational processing for MSSM donors” sections. We made use of the existing genotyping data from MSSM to assess genotype concordance with the newly generated SNP array. This allowed us to identify sample swaps and unintended duplicates. Lastly, we implemented a multi-step procedure to merge genotypes from the existing and newly generated WGS and SNP array data, resulting in the creation of a comprehensive genotype dataset for the PsychAD cohort, as outlined in the “Integration of multi-source genotype data” section.

#### SNP arrays

Genomic DNA was isolated using the QIAamp DNA mini kit (Qiagen), according to the manufacturer’s instructions, and quantified via Qubit (Life Technologies). 400 ng of DNA was then whole-genome amplified, fragmented, precipitated and resuspended in the appropriate hybridization buffer. Genotyping was performed using the Infinium Global Screening Array-24 Kit (Illumina) according to the manufacturer’s protocol. Briefly, denatured samples were hybridized on prepared Illumina Bead Chips. After hybridization, the Bead Chip oligonucleotides were extended by a single fluorescent labeled base, which was detected by fluorescence imaging with an Illumina Bead Array Reader, iScan.

#### Computational processing for MSSM donors

Pre-imputation processing of the PsychAD MSSM subcohort genotype data consisted of running the quality control script HRC-1000G-check-bim.pl from the McCarthy Lab Group (https://www.well.ox.ac.uk/~wrayner/tools/). Genotypes were then phased and imputed on the Trans-Omics for Precision Medicine (TOPMed) Imputation Server^62^. Only variants with an imputation R2 > 0.3 were retained. Biallelic variants were additionally annotated with ancestry-specific MAF values from the National Center for Biotechnology Information’s Allele Frequency Aggregator (ALFA) (https://ftp.ncbi.nih.gov/snp/population_frequency/latest_release/). The populations included in the ALFA database are described at https://www.ncbi.nlm.nih.gov/snp/docs/gsr/data_inclusion/#population.

#### Integration of multi-source genotype data

To compile a combined dataset covering the largest possible fraction of the PsychAD cohort, we leveraged the following datasets:

- PsychAD-MSSM SNP array: Genotyping data for 882 donors that cover the majority of MSSM donors were generated and described in this manuscript.
- CommonMind SNP array: Genotyping data for 513 samples overlapping the PsychAD cohort were obtained from previously generated SNP array on Illumina Infinium HumanOmniExpressExome 8 v 1.1b chip^33^. These data are accessible to all registered users of the NIMH Data Archive (RRID:SCR_004434) under the collection identifier C5063.
- RADC WGS: Whole-genome sequencing data for 131 samples overlapping the PsychAD cohort were obtained from a Diverse study^61^ available to all registered users of the AD Knowledge Portal (RRID:SCR_006307) under the release number 20.6, accession identifier syn51757644.
- ADSP WGS: Whole-genome sequencing data for 284 samples overlapping the PsychAD cohort were obtained from the seventh release of WGS data under the ADSP Umbrella Study (NG00067.v7) from the National Institute of Aging Genetics of Alzheimer’s Disease Data Storage Service^63^.

**Fig. 3:**
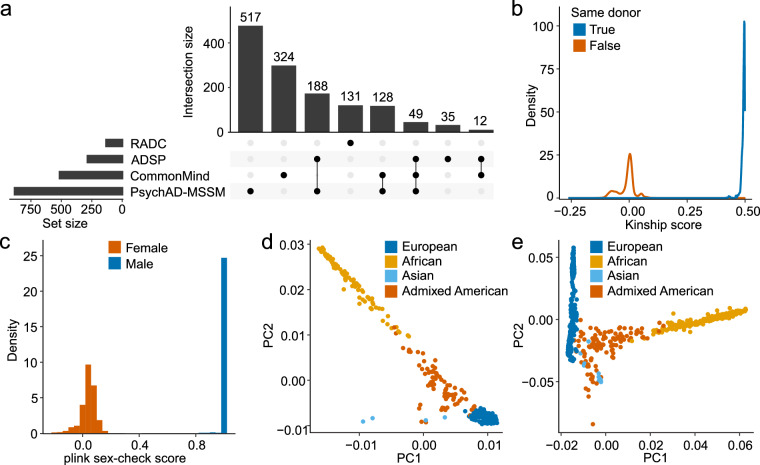
Analysis of genotyping data. (**a**) Counts and intersections among sources of genotyping data available for donors from the PsychAD cohort. (**b**) Distribution of genetic similarities estimated between combined genotype dataset and genotypes called from snRNA-seq data. (**c**) F-statistic from plink’s “check-sex” function plotted by reported sex (samples with known sex chromosome aneuploidies not shown). (**d,e**) The first two PCs of genetic ancestry were calculated separately for the PsychAD-MSSM genotype dataset of 882 samples (**d**) and for the combined dataset of 1,381 samples (**e**).

These datasets exhibited a significant overlap, resulting in 377 donors with one or more duplicates, as identified by KING^64^ (kinship score ≥ 0.4; Fig. 3a). To determine which of the duplicated samples were retained for use as the final genotype file, the following criteria were applied: First, if one sample was obtained through WGS and the other through SNP array genotyping, we retained the WGS sample. This prioritization criterion was applied to 249 donors. Next, if one sample displayed a heterozygosity value falling within ± 2 standard deviations from the mean, we retained the other sample, thus excluding an additional 8 pairs. All of the remaining 120 donors had samples in both the PsychAD-MSSM and CommonMind SNP arrays. We opted to prioritize the PsychAD-MSSM SNP array due to inherent imperfections of the CommonMind SNP array, which was initially provided in hg19 coordinates and necessitated conversion to hg38 coordinates. As a result, the final combined genotype file encompasses 92% (1,381) of the PsychAD donors.

Whole-genome sequencing (WGS) variant calling for the RADC and ADSP samples was carried out according to the best practice guidelines of the Genome Analysis Toolkit (GATK)^65^. In summary, the identification of single nucleotide variants (SNVs) and insertions/deletions (indels) was performed jointly using GATK’s HaplotypeCaller and GenotypeGVCFs tools. The refinement and annotation of variants were achieved through Variant Quality Score Recalibration (VQSR) within the GATK environment. Quality control (QC) steps followed previously established pipelines^66–68^. For sample-level QC, relatedness, DNA contamination, and sample-level missingness (samples excluded if > 0.05) were evaluated, as well as overall coverage (samples excluded if < 25x). Outlier samples were identified and excluded based on several criteria, including the number of called SNVs and indels, insert size length, alignment mapping quality score (MQ), CRAM file size, transition/transversion ratio (Ti/Tv), the ratio of novel variants to all variants, and the ratio of mapped reads to paired reads, as detailed in previous studies^66–68^. At the variant level, filtering removed variants with more than 10% missingness and high levels of heterozygosity (InbreedingCoeff <−0.8). Individual genotype calls with a depth (DP) of less than 10 or a genotype quality (GQ) of less than 20 were marked as missing. Analyses were limited to biallelic variants only.

#### Ancestry estimation

Based on the success of Mahalanobis distance techniques in ancestry assignment^69,70^, we leveraged quadratic discriminant analysis (QDA) to assign ancestry using scikit-learn^71^. We determined the genetic ancestry of our samples based on the five superpopulations defined by the 1000 Genomes Project. Initially, we merged unimputed genotypes with the 1000 Genomes Project data on the GRCh38 v2a reference using BCFtools version 1.9. We then computed the principal components (PCs) of the merged genotypes using PLINK PCA. The merged genotype used for this calculation was variant-level filtered to keep only single nucleotide variants (SNVs) with a minor allele frequency (MAF) of at least 0.01, a Hardy-Weinberg equilibrium (HWE) P-value of at least 10^−10^, and a variant-level missingness of no more than 0.01. We also performed linkage disequilibrium (LD) pruning with a window size of 1,000 kb, a step size of 10, and an R^2^ threshold of 0.2. Finally, we used forward selection to choose PC1 through PC6 for training the QDA models, applying a regularization parameter of 5^−7^.

## Data Records

Raw and processed data described herein are available for use by the research community and have been deposited in the AMP-AD Knowledge Portal in the study-specific folder^72^. These include sample metadata, as well as raw and processed sequencing data for snRNA-seq and genotyping. Single nuclei data can be inspected at the CELLxGENE (RRID:SCR_021059) portal at https://cellxgene.cziscience.com/collections/84ce6837-548d-4a1f-919f-0bc0d9a3952f.

## Technical Validation

### Genotype data quality control

Out of four genotype datasets used in this study, three external datasets already underwent QC before they were released so we performed only a limited check. For the newly generated PsychAD-MSSM SNP array data, we started by removing samples with missingness over 0.05 (calculated within a subset of high-quality variants with variant-level missingness ≤ 0.02). All SNP-array samples were compared against genotypes called from snRNA-seq samples to check the across-assay concordance for samples originating from the same donor. This comparison allowed us to resolve sample swaps in both assays, as well as to detect and remove duplicated and contaminated samples (Fig. 3b). Next, the samples with a mismatch between the self-reported and genetically inferred sex were removed, as well as those with outlier heterozygosity defined by ± 3 standard deviations from the mean (samples with known sex chromosome aneuploidies were not subjected to this check). After performing all QC steps, we observed unambiguous separation of male and female samples and good concordance of inferred and reported ancestry for all remaining 882 samples (Fig. 3c,d). Comparable results were obtained for the merged genotype dataset, which included 1,381 samples (Fig. 3e).

### snRNA-seq data quality control

**Fig. 4:**
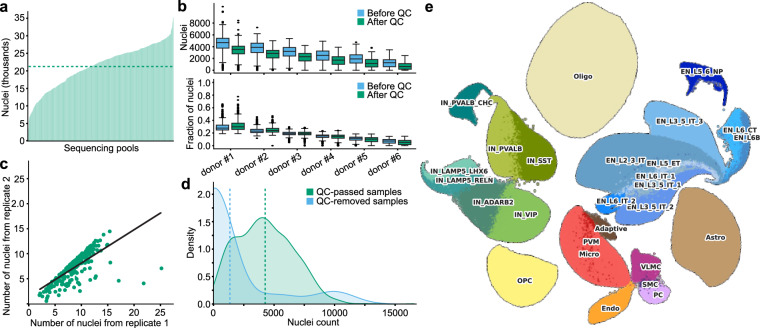
Analysis of the snRNA-seq dataset. (**a**) Distribution of the number of nuclei across sample pools. Dashed line indicates the mean. (**b**) Distribution of nuclei to libraries within pools, ordered by nuclei count (top) and fraction of nuclei (bottom). Each replicate is depicted using two boxplots representing the nuclei distribution before (blue) and after QC (green). The center line (black) indicates the median, the box shows the interquartile range, and the whiskers indicate the highest/lowest values within 1.5 × the interquartile range. **(c)** Comparison of QC-passed nuclei counts between pairs of replicates from the same sequencing pools (Spearman’s ρ = 0.84). (**d**) Distribution of nuclei counts in samples that passed or failed QC (vertical line indicates the mean values). (**e**) UMAP visualization of snRNA-seq data. IN: inhibitory/GABAergic neurons, EN: excitatory/glutamatergic neurons, SMC: smooth muscle cells, VLMC: vascular leptomeningeal cells, PVM: perivascular macrophages, OPC: oligodendrocyte progenitor cells, Astro: astrocytes, Oligo: oligodendrocytes, Micro: Microglia, Endo: endothelial, Adaptive: adaptive immune cells, PC: Pericytes.

After the QC processes, our snRNA-seq dataset consisted of 6.32 million nuclei spread across 561 pools. Each pool contained six libraries, and each library underwent sequencing in duplicate on different sequencing lanes. The typical yield was about 21,238 nuclei per pool (Fig. 4a), which were anticipated to be distributed evenly across the libraries. Nevertheless, we observed considerable variability in nuclei counts, largely attributed to variances in tissue quality that impact cell viability and capture efficiency^73^. The largest replicate in a typical pool accounted for about 32% of the nuclei (3,367 nuclei), while the smallest only captured about 5% (721 nuclei) (Fig. 4b**)**. Such fluctuations are not unusual and align with findings from other studies^53,73,74^. Despite these discrepancies in nuclei numbers, the replicates consistently showed a high correlation in gene expression signals (Spearman’s ρ = 0.82), underscoring the data’s robustness (Fig. 4c). Notably, samples discarded at the QC step had roughly 55% fewer nuclei than those that met the QC criteria (1,920 vs. 4,240 nuclei, Fig. 4d). The cellular taxonomy revealed eight major cell classes and 27 subclasses (Fig. 4e).

## Usage Notes

**Fig. 5:**
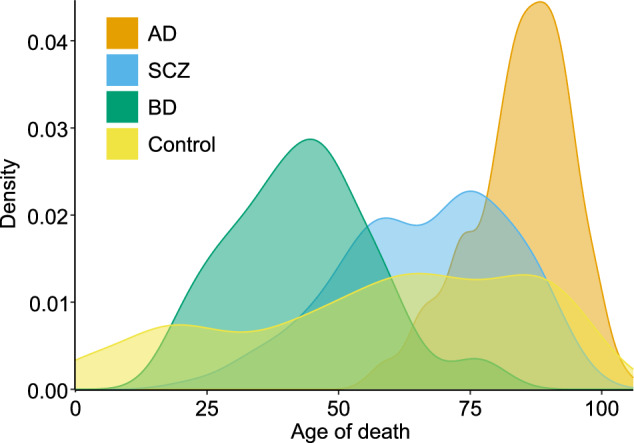
Distribution of the age at death stratified by diagnosis. The diagnoses shown in this plot were intentionally selected to highlight age differences.

Having a dataset with such a large scope, including over 6 million nuclei, 1,494 donors, 33 diagnoses, and ages ranging from 0 to 108 years, presents many opportunities but also demands careful handling. A common use case involves a statistical comparison of two groups of donors, typically those from disease carriers and neurotypical controls. In such scenarios, it is important to ensure the careful selection of donors for the control group because controls have typically much lower age at the time of death in our dataset (Fig. 5). Due to the impact of normal aging on cell function^11^, a wide variation in the age distributions of the groups being compared can obscure the actual effects of the disease. While complex non-linear modeling can adjust for some of these effects in differential analysis, we still recommend setting a minimum age threshold. In disease-oriented papers published using this dataset^23–25,28,29^, we established a minimum age of 17 years for serious mental illnesses and 60 years for neurodegenerative disorders. While researchers can choose which effects they want to correct for in their analysis, it’s worth noting that our studies typically adjust for demographic factors such as sex, brain bank, and postmortem interval, each of which was modeled as having a linear effect.

All data are available via the AD Knowledge Portal (https://adknowledgeportal.org). The AD Knowledge Portal is a platform for accessing data, analyses, and tools generated by the Accelerating Medicines Partnership (AMP-AD) Target Discovery Program and other National Institute on Aging (NIA)-supported programs to enable open-science practices and accelerate translational learning. The data, analyses and tools are shared early in the research cycle without a publication embargo on secondary use. Data is available for general research use according to the following requirements for data access and data attribution (https://adknowledgeportal.synapse.org/Data%20Access).

## Data Availability

The source code used to analyze the metadata and create figures for this manuscript can be found on GitHub at this location: https://github.com/DiseaseNeuroGenomics/psychAD_SciData.
